# The Prognostic Significance of Soluble Urokinase Plasminogen Activator Receptor in Acute Myeloid Leukemia

**DOI:** 10.4274/tjh.2014.0405

**Published:** 2016-05-16

**Authors:** Nergiz Erkut, Ahmet Menteşe, Hasan Mücahit Özbaş, Nilay Ermantaş, Ayşegül Sümer, Asım Örem, Mehmet Sönmez

**Affiliations:** 1 Kanuni Training and Research Hospital, Clinic of Hematology, Trabzon, Turkey; 2 Karadeniz Technical University Faculty of Medicine, Department of Hematology, Trabzon, Turkey; 3 Karadeniz Technical University, Vocational School of Health Sciences, Program of Medical Laboratory Techniques, Trabzon, Turkey; 4 Karadeniz Technical University Faculty of Medicine, Department of Medical Biochemistry, Trabzon, Turkey

**Keywords:** Soluble urokinase plasminogen activator receptor, Acute myeloid leukemia, prognosis

## Abstract

**Objective:**

The soluble urokinase plasminogen activator receptor (suPAR) is a soluble form of the urokinase plasminogen activator receptor expressed in various immune and cancer cells. The levels of suPAR have been demonstrated to correlate with prognosis in various cancers. This study was intended to investigate serum suPAR levels and their effect on prognosis in patients with acute myeloid leukemia (AML).

**Materials and Methods:**

Thirty newly diagnosed patients with AML and 29 healthy individuals were enrolled. Serum suPAR levels were analyzed by enzyme-linked immunosorbent assay.

**Results:**

Serum suPAR levels were significantly higher in patients with AML than in healthy individuals (9±5.9 ng/mL and 2.4±1.4 ng/mL, respectively; p<0.001). Positive correlation was determined between suPAR levels and white blood cell counts (p<0.01). Serum suPAR levels were lower in patients who achieved complete response than in patients not achieving complete response (5.5±2.2 ng/mL and 12±6.6 ng/mL, respectively; p<0.001). The median overall survival was longer in patients with serum suPAR levels below 6.71 ng/mL than in those with serum suPAR levels above 6.71 ng/mL (12.6±13.2 months and 1.71±0.6 months, respectively; p=0.02). Multivariate Cox regression analysis showed that suPAR had independent prognostic value (95% confidence interval: 1.029-6.259; p<0.05) in AML.

**Conclusion:**

Serum suPAR levels can be used as a prognostic marker in AML.

## Abstract (tr)

**Amaç:**

Solubl ürokinaz plazminojen aktivatör reseptörü (süPAR) çeşitli immün sistem ve kanser hücrelerinde eksprese edilen ürokinaz plazminojen aktivatör reseptörün çözünür formudur. Çeşitli kanserlerde süPAR düzeyinin prognoz ile ilişkili olduğu gösterilmiştir. Bu çalışmada akut miyeloid lösemili (AML) hastalarda süPAR düzeyi ve prognoz üzerine olan etkisinin araştırılması planlandı.

**Gereç ve Yöntemler:**

Çalışmaya yeni tanı almış 30 AML’li hasta ve 29 sağlıklı birey dahil edildi. Serum süPAR düzeyi enzyme-linked immunosorbent assay yöntemi ile analiz edildi.

**Bulgular:**

Serum süPAR düzeyi AML’li hastalarda sağlıklı bireylere göre önemli derecede daha yüksek tespit edildi (9±5,9 ng/mL, 2,4±1,4 ng/mL, sırasıyla, p<0,001). süPAR düzeyi ile lökosit sayısı arasında pozitif bir korelasyon izlendi (p<0,01). Serum süPAR düzeyi, tam remisyona giren hastalarda tam remisyona girmeyen hastalara göre daha düşüktü (5,5±2,2 ng/mL, 12±6,6 ng/mL, sırasıyla, p<0,001). Toplam yaşam süresi, serum süPAR düzeyi 6,71 ng/mL’nin altında olan hastalarda, 6,71 ng/mL üstünde olanlara göre daha uzundu (12,6±13,2 ay, 1,71±0,6 ay, sırasıyla, p=0,02). AML’de çok değişkenli Cox regresyon analizi süPAR düzeyinin bağımsız prognostik değere sahip olduğunu gösterdi (%95 güven aralığı: 1,029-6,259; p<0,05).

**Sonuç:**

AML’li hastalarda serum süPAR düzeyi prognostik bir belirteç olarak kullanılabilir.

## INTRODUCTION

Acute myeloid leukemia (AML) is a heterogeneous neoplastic disorder characterized by uncontrolled proliferation of hematopoietic stem cells [1]. Although 70%-80% of patients younger than 60 years of age achieve complete remission (CR), only 30%-40% obtain long-term survival. Moreover, CR is only observed in 10%-15% of elderly patients [2]. The pathogenesis of AML involves various disorders, such as mutations in transcription factors or epigenetic modifiers, aberrant signaling pathways, overexpression of the multidrug resistance gene, abnormal immune function, and abnormalities in the bone marrow microenvironment [3]. Prognostic factors include advanced age, poor performance status, high white blood cell (WBC) count, existence of prior myelodysplastic syndrome and myeloproliferative disease, previous history of cytotoxic therapy, and particularly cytogenetics and molecular genetic changes [4,5].

The urokinase plasminogen activator receptor (uPAR) is a glycoprotein consisting of 274 amino acids with a molecular weight of 55-60 kDa attached to the plasma membrane via a glycosylphosphatidylinositol anchor protein [6]. uPAR is expressed in neutrophils, lymphocytes, monocytes, macrophages, fibroblasts, and endothelial and some tumor cells [7,8,9]. The soluble urokinase plasminogen activator receptor (suPAR) is a soluble form of uPAR found in serum, plasma, urine, and other body fluids [10]. suPAR affects cancer progression through adhesion, migration, chemotaxis, proteolysis, and invasion [11]. Several studies have demonstrated that suPAR increases in some cancers and is associated with poor prognosis [12]. This study was intended to investigate serum suPAR levels and their effect on prognosis in patients with AML.

## MATERIALS AND METHODS

Thirty newly diagnosed patients with AML and 29 healthy individuals presenting to the Deparment of Hematology, Faculty of Medicine, Karadeniz Technical University between January 2009 and July 2011 were enrolled in this study. The eligibility criterion was age between 18 and 80 years. Patients with a history of solid cancer or other hematological cancer, the presence of active infection, or active inflammatory disease were excluded. Venous blood specimens collected from both patient and control groups were placed into biochemical separator-containing tubes. Blood samples were centrifuged at 3000 rpm for 10 min and serum was stored at -80 °C for investigation of suPAR levels.

All AML patients were diagnosed according to the World Health Organization classification system [13] and categorized into three groups (i.e. low risk, intermediate risk, and high risk) according to the National Comprehensive Cancer Network guidelines [14].

Patients aged ≤60 years or 61-65 years with good performance status were treated with the standard regimen [cytarabine, 24-h continuous intravenous (IV) infusion, 100 mg/m2, days 1-7; idarubicin, 30-min IV infusion, 12 mg/m2, days 1-3]. Patients with acute promyelocytic leukemia were treated with all-trans-retinoic acid (ATRA) plus idarubicin therapy (ATRA, orally, 45 mg/m2 per day in two divided doses until CR was achieved; idarubicin, 30-min IV infusion, 12 mg/m2, days 2, 4, 6, and 8). Elderly patients were treated with low-dose chemotherapy [low-dose cytarabine, subcutaneous (SC), 10 mg/m2, twice a day, days 1-10; or 5-azacytidine, SC, 75 mg/m2, days 1-7]. Remission status was evaluated after the completion of cancer therapy according to conventional criteria. Patients were followed for 2 years, monthly for the first year and every third month in the following year.

### Measurement of Soluble Urokinase Plasminogen Activator Receptor Levels

The levels of serum suPAR were determined by enzyme-linked immunosorbent assay kit (ViroGates A/S, Denmark) according to the manufacturer’s protocols. The absorbance of samples was measured at 450 nm using a VERSA max tunable microplate reader (designed by Molecular Devices, USA). The results were expressed as ng/mL. The minimum detection limit of the assay was estimated to be 0.1 ng/mL.

### Statistical Analysis

All analyses were carried out using SPSS 21.0. Descriptive statistical analysis was performed for all studied variables. Data were tested for normal distribution using the Kolmogorov-Smirnov test. Statistical comparisons between the patient and control groups were carried out using the Mann-Whitney test and chi-square test. The associations between serum suPAR levels and hemoglobin (Hb) or hematocrit levels and white blood cell (WBC) or platelet count were examined by Spearman correlation analysis. The area under the receiver operating characteristic (ROC) curve was used to compare the discriminative power of suPAR levels in the diagnosis of AML. Estimates of overall survival (OS) were calculated using the Kaplan-Meier method. The log-rank test was used to analyze the effect on survival time of each variable. The Cox regression model was applied for multivariate analysis. Linear regression analysis was used to investigate the relationship between serum suPAR levels and sex, patient age, WBC count, French-American-British (FAB) classification, and Fms-like tyrosine receptor kinase-3 (FLT-3) mutation. A value of p<0.05 was considered statistically significant.

## RESULTS

Thirty patients with AML and 29 healthy controls were included in the study. There were no statistical differences in term of age or sex between the two groups. Risk groups included 6 patients at good risk, 19 at intermediate risk, and 5 at poor risk. At the end of the 2-year follow-up, 26 patients had died and 4 survived. Fourteen patients exhibited CR after remission-induction chemotherapy, while CR was not achieved in the other 16. Table 1 shows the general characteristics and laboratory findings for both patients and healthy individuals.

**Figure 1:**
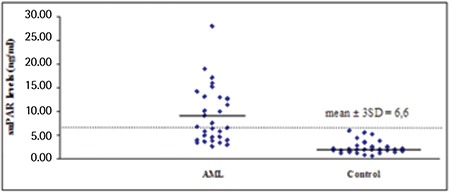
Soluble urokinase plasminogen activator receptor concentrations in serum from acute myeloid leukemia patients and healthy controls. The dotted line indicates the mean value plus 3 standard deviations of healthy control serum (6.6 ng/mL). suPAR: Soluble urokinase plasminogen activator receptor, AML: acute myeloid leukemia, SD: standard deviation.

**Figure 2:**
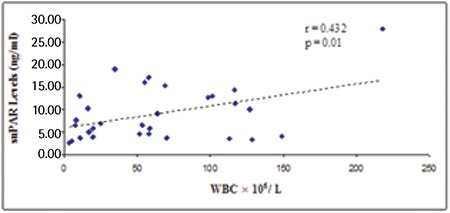
Correlations between soluble urokinase plasminogen activator receptor levels and white blood cell count in acute myeloid leukemia patients. WBC: White blood cell, suPAR: soluble urokinase plasminogen activator receptor, AML: acute myeloid leukemia.

**Figure 3:**
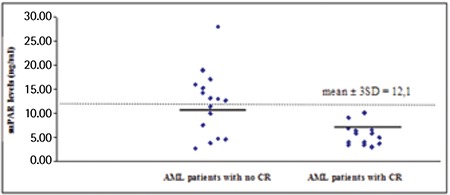
Soluble urokinase plasminogen activator receptor concentrations in serum from acute myeloid leukemia patients with no complete remission and acute myeloid leukemia patients with complete remission. The dotted line indicates the mean value plus 3 standard deviations of serum of acute myeloid leukemia patients with complete remission (12.1 ng/mL). suPAR: Soluble urokinase plasminogen activator receptor, AML: acute myeloid leukemia, SD: standard deviation, CR: complete remission.

Serum suPAR levels were significantly higher in patients with AML than in healthy individuals (9±5.9 ng/mL and 2.4±1.4 ng/mL, respectively; p<0.001) (Figure 1). Positive correlation was determined between suPAR levels and WBC count in patients with AML (p<0.01) (Figure 2), whereas there was no correlation between suPAR levels and Hb levels or platelet count. There was no significant difference in serum suPAR levels between patients aged ≤60 and >60 years (7.6±4.4 ng/mL and 12.3±8 ng/mL, respectively; p>0.05). Serum suPAR levels were lower in patients who achieved CR than in patients not achieving CR (5.5±2.2 ng/mL and 12±6.6 ng/mL, respectively; p<0.001) (Figure 3).

**Figure 4:**
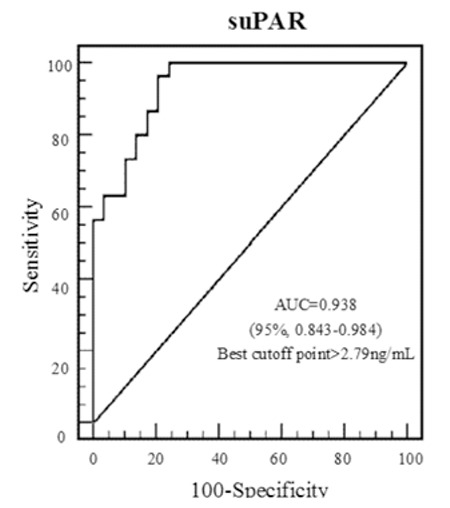
The receiver operating characteristic curves of acute myeloid leukemia patients according to soluble urokinase plasminogen activator receptor levels. suPAR: Soluble urokinase plasminogen activator receptor, AUC: area under the curve.

In AML patients, the area under the ROC curve for suPAR was 0.938 [95% confidence interval (CI): 0.843-0.984]. For the optimum diagnostic cut-off value of 2.79 ng/mL, the sensitivity and specificity were 96.67% and 79.31%, respectively (Figure 4).

**Figure 5:**
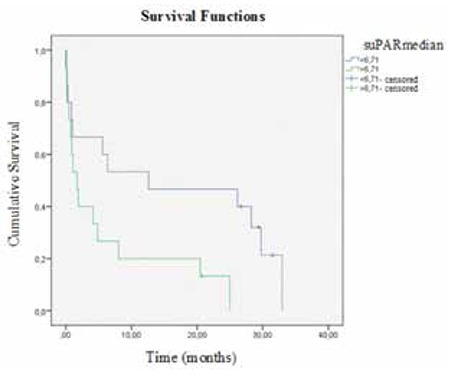
Kaplan-Meier curves of acute myeloid leukemia patients according to soluble urokinase plasminogen activator receptor levels. suPAR: Soluble urokinase plasminogen activator receptor.

**Figure 6:**
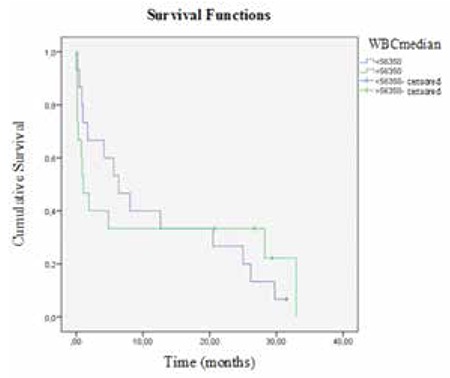
Kaplan-Meier curves of acute myeloid leukemia patients according to white blood cell count. WBC: White blood cell.

The median OS of AML patients was 4.16 months (range: 0-32.9 months). In the Kaplan-Meier analysis and the Cox regression model, patients with high serum suPAR levels showed a trend toward poorer survival (p=0.02). The median OS was longer in patients with serum suPAR levels below 6.71 ng/mL than in those with serum suPAR levels above 6.71 ng/mL (12.6±13.2 months and 1.71±0.6 months, respectively; p=0.02) (Figure 5). WBC count had no significant effect on OS (p=0.9) (Figure 6). In linear regression analysis, sex, patient age, WBC count, FAB classification (i.e. M2, M3, M4, M5), and FLT-3 mutation were not associated with serum suPAR levels (p>0.05). Multivariate Cox regression analysis showed that suPAR had independent prognostic value (95% CI: 1.029-6.259; p<0.05) in AML. When the suPAR cut-off level was considered as 6.71 ng/mL, mortality risk was 2.5-fold higher in patients with levels above the cut-off limit.

## DISCUSSION

The urokinase-mediated plasminogen activation (uPA) system plays an important role in tissue remodeling, angiogenesis, proteolysis, migration, chemotaxis, invasion, and metastasis [15,16,17]. The uPA system consists of uPA, uPAR, plasminogen, and plasminogen activator inhibitor [18]. In vitro studies have shown that suPAR is associated with cell adhesion, migration, and proliferation [19,20]. Elevated suPAR levels have been determined in solid cancers such as ovarian [21], endometrial, cervical [22], breast [23], stomach [24], colon [25], and non-small cell lung cancer [26]. Positive associations between serum suPAR levels and soluble serum CD138, creatinine, β2 microglobulin, stage of disease, and extramedullary bone marrow involvement have been reported in patients with multiple myeloma [27].

In acute leukemia, circulating blast cells provide an important advantage for studying proteins expressed on the tumor cell surface. Lanza et al. demonstrated that uPAR (CD87) expression increased in patients with AML and was associated with mucocutaneous infiltration, hepatosplenomegaly, lymphadenopathy, and central nervous system involvement [28]. The levels of suPAR in the plasma of mice during the growth of xenografted cell lines were significantly related to tumor volume [29]. Mustjoki et al. reported that increased suPAR levels were correlated with number of circulating tumor cells in AML and that serum suPAR levels decreased rapidly after cytotoxic treatment [30]. Aref et al. further demonstrated that serum suPAR levels were significantly higher in AML patients as compared to controls [31]. Similarly, in our study, serum suPAR levels significantly increased in patients with AML compared to healthy individuals. In addition, suPAR was observed to possess high sensitivity and speciﬁcity in patients with AML in ROC analysis. There was a positive correlation between suPAR levels and number of circulating WBCs. Therefore, we think that the production of suPAR is related to blast cells in the peripheral circulation.

Lomholt et al. demonstrated that elevated suPAR levels were independent prognostic factors in patients with colorectal cancer [32]. Another study showed that high suPAR levels were associated with poor outcome in patients with breast cancer independent of tumor size, estrogen receptor status, and lymph node status [23]. On the other hand, Begum et al. reported that preoperative plasma suPAR levels were not correlated with prognosis for stage III ovarian cancer patients [33]. In our study, serum suPAR levels were significantly higher in patients who did not achieve CR than in patients achieving CR. More importantly, high suPAR levels were associated with poor prognosis in patients with AML. When the suPAR cut-off level was considered as 6.71 ng/mL, mortality risk was 2.5-fold higher in patients with levels above the cut-off limit. Sex, patient age, WBC count, FAB classification (i.e. M2, M3, M4, M5) and FLT-3 mutation were not associated with serum suPAR levels (p>0.05). Serum suPAR levels were an independent prognostic indicator for the OS of patients with AML.

## CONCLUSION

In conclusion, our study indicates that suPAR increases in patients with AML and this situation is associated with poorer survival. suPAR can thus be used as a diagnostic and prognostic biomarker in AML and may help in the developing of specific therapeutic targets. However, further studies are required to assess the clinical relevance of suPAR.

## Ethics

Ethics Committee Approval: The study was approved by the Local Ethics Committee of the Karadeniz Technical University Faculty of Medicine, and was conducted in accordance with the Declaration of Helsinki. Informed consent was taken from all patients and healthy subjects.

## Figures and Tables

**Table 1:**
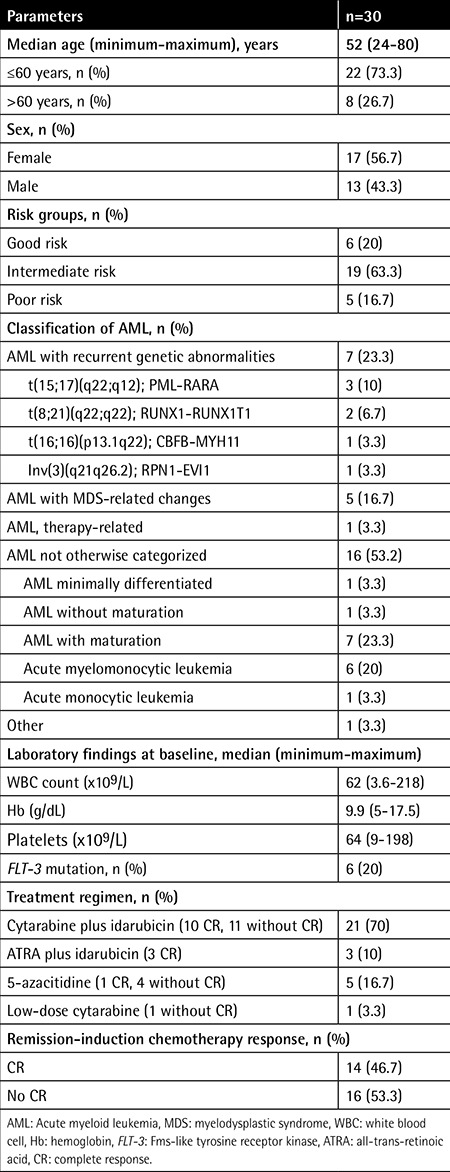
Characteristics of acute myeloid leukemia patients.
